# A pilot study of evaluation of semi-rigid and flexible catheters for less invasive surfactant administration in preterm infants with respiratory distress syndrome—a randomized controlled trial

**DOI:** 10.1186/s12887-022-03714-3

**Published:** 2022-11-04

**Authors:** Lorenz Auer-Hackenberg, Johannes Brandner, Edda Hofstätter, Patricia Stroicz, Tobias Hager, Anna Eichhorn, Sebastian Schütz, Raphael Feldner, Martin Wald

**Affiliations:** 1 Division of Neonatology, Department of Pediatrics and Adolescent Medicine, Paracelsus Medical University, Müllner-Haupt-Str. 48, 5020 Salzburg, Austria; 2 Clinical Research Center Salzburg, Salzburg, Austria

**Keywords:** Less Invasive Surfactant Application, Minimal Invasive Surfactant Application, Preterm Infants, Respiratory Distress Syndrome

## Abstract

**Background:**

In respiratory distress syndrome, many neonatology centers worldwide perform minimal invasive surfactant application in premature infants, using small-diameter catheters for endotracheal intubation and surfactant administration.

**Methods:**

In this single-center, open-label, randomized-controlled trial, preterm infants requiring surfactant administration after birth, using a standardized minimal invasive protocol, were randomized to two different modes of endotracheal catheterization: Flexible charrière-4 feeding tube inserted using Magill forceps (group 1) and semi-rigid catheter (group 2). Primary outcome was duration of laryngoscopy. Secondary outcomes were complication rate (intraventricular hemorrhage, soft-tissue damage in first week of life) and vital parameters during laryngoscopy. Between 2019 and 2020, 31 infants were included in the study. Prior to in-vivo testing, laryngoscopy durations were studied on a neonatal airway mannequin in students, nurses and doctors.

**Results:**

Mean gestational age and birth weight were 27 + 6/7 weeks and 1009 g; and 28 + 0/7 weeks and 1127 g for group 1 and 2, respectively. Length of laryngoscopy was similar in both groups (61.1 s and 64.9 s) overall (p.77) and adjusted for weight (p.70) or gestational age (p.95). Laryngoscopy failed seven times in group 1 (43.8%) and four times (26.7%) in group 2 (p.46). Longer laryngoscopy was associated with lower oxygen saturation with lowest levels occurring after failed laryngoscopy attempts. Secondary outcomes were similar in both groups. In vitro data on 40 students, 40 nurses and 12 neonatologists showed significant faster laryngoscopy in students and nurses group 2 (*p* < .0001) unlike in neonatologists (p.13).

**Conclusion:**

This study showed no difference in laryngoscopy duration in endotracheal catheterization when comparing semi-rigid and flexible catheters for minimal invasive surfactant application in preterm infants. In accordance with preliminary data and in contrast to published in-vitro trials, experienced neonatologists were able to perform endotracheal catheterization using both semi-rigid and flexible catheters at similar rates and ease, in vitro and in vivo.

**Trial registration:**

ClinicalTrials.gov. NCT05024435 Registered 27 August 2021—Retrospectively registered.

**Supplementary Information:**

The online version contains supplementary material available at 10.1186/s12887-022-03714-3.

## Background

In neonatology, the administration of exogenous surfactant into the lungs of premature newborns with respiratory distress syndrome (RDS) is a well-recognized and important tool to increase survival, especially in extremely low birth weight (ELBW) infants, and decrease neurologic morbidity (such as intracranial haemorrhage) and chronic lung disease (such as broncho-pulmonary dysplasia, BPD) [1, 2]. Today, surfactant is one of the cornerstones in the therapy of RDS in newborns. Its application has been extensively researched and a variety of surfactant delivery methods now exists [3–8].

### Minimal invasive methods for surfactant application

The application of continuous positive airway pressure (CPAP), particularly immediately after birth is another important factor during resuscitation of extremely preterm babies. Studies showed that RDS is a risk factor for CPAP failure. These studies finally led the way to surfactant application in the delivery room [9]. The optimal mode and time of surfactant delivery into the respiratory tract is a matter of different investigations. Minimal invasive surfactant treatment (MIST) techniques, also known as less invasive surfactant application (LISA), offer the possibility to administer surfactant in spontaneously breathing infants, who are on CPAP respiratory support, using a small intratracheal catheter and thereby avoiding positive pressure ventilation, endotracheal intubation and general anaesthesia [5, 6, 10, 11]. Recently, the European Society for Paediatric Research published recommendations on postnatal infant care and surfactant application in premature infants with RDS [8]. These guidelines are based on findings which suggest LISA’s superiority in terms of reduction in death, mechanical ventilation and BPD. When compared to other surfactant application via endotracheal tubes, LISA is the preferred method of trained neonatologists for spontaneously breathing infants on CPAP [7, 8, 10, 12–16].

Despite wide acceptance of LISA as a standardized protocol for surfactant, discussion remains about application and adoption of this technique in many centers worldwide. The optimal catheter to use for intubation and surfactant administration is still subject to debate [5]. Preliminary data underlines the advantages of rigid catheters over flexible feeding tubes [6, 17, 18]. Most recently, Rigo and colleagues showed that the insertion of stylet-guided catheters or rigid tubes is significantly faster than that of flexible feeding tubes (inserted with the help of Magill forceps) during intubation of neonatal airway dummies [18]. However, most of these studies solely compare catheters in airway simulators (e.g. manequins of preterm infants). In these studies, neonatologists perform minimal invasive endotracheal catheterization with different devices in video recorded simulations and the footage is analyzed in terms of correct tube placement and procedural duration [18]. Currently to day, due to lacking evidence in vivo and in vitro, the choice of the appropriate surfactant administration device is most oftenly based on doctors’ personal preferences, local organizational peculiarites, or catheter availability.

**Fig. 1:**
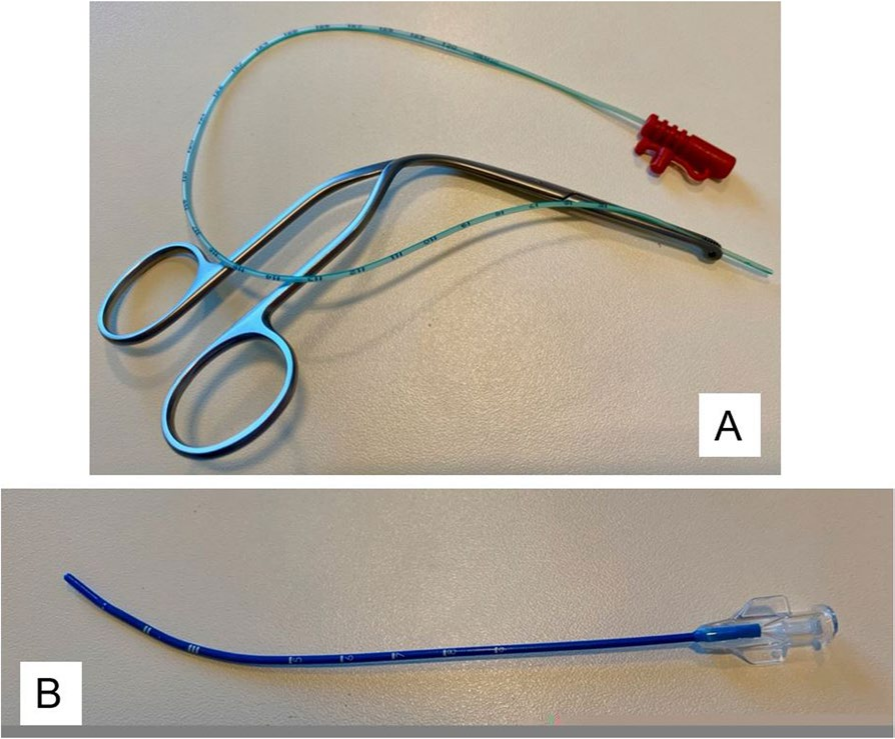
Catheters for surfactant administration (our own photo). **A** Nasogastric tube (Unomedical Charrière 4; outer diameter 1.3 mm; inner diameter 0.66 mm; length 375 mm flexible REF 12,021,182, ConvaTec UK) and magill forceps. **B** LISAcath® (1.7 × 130 mm REF 145,872–01 Chiesi Farmaceutici S.p.a. Italy) slightly bent to facilitate intubation

Since 2007, our centre has been using LISA as a standardized mode of surfactant application during initial resuscitation of extremely premature infants born prior to 28 weeks of gestation or in infants born less than 32 weeks of gestation with early clinical signs of RDS (i.e. FiO2 > 0.3 on CPAP of at least 6cmH2O). We currently use both flexible feeding tubes and rigid catheters for intratracheal surfactant delivery (Fig. 1) (video at: https://www.youtube.com/watch?v=0OmXlOXETZY) [10]. This study aims to investigate if there are advantages of one technique over the other in vivo as suggested by in vitro results [18].

## Materials and methods

This study was designed as an open-label randomized controlled study at the Neonatology Division (University Hospital Salzburg, Paracelsus Medical Private University). Preterm infants admitted to our department who required surfactant administration using LISA protocol were enrolled prior to birth, once parental informed consent was obtained (see supplementary material on LISA protocol).

### Preliminary data

This study was separated into two parts. The initial experiments (February 2019), sought to gather preliminary data on the ability and efficiency of untrained medical students, neonatal intensive care unit (NICU) nurses and neonatologists (with at least three years of training) to administer surfactant using both semi rigid catheter for surfactant administration (LISAcath®; Chiesi Farmaceutici S.p.a. Italy) and a conventional nasogastric tube. Participants were placed in groups of four to six. Nurses and students, were given ten minutes of basic introduction, in which both techniques were demonstrated using a video laryngoscope and the preterm baby simulation dummy “Paul” (SIMCharacters GmbH, Vienna). “Paul” is a state-of-the-art preterm baby simulator representing an infant born at 27 + 3/7 weeks of gestation and weighing 1000 g. After the introduction, all participants had to successfully intubate the dummy using both techniques. During training, participants were supervised and coached by the investigators.

After a total of 50 min of this training, we used a block-randomization of four to randomly allocated students and nurses to one technique. Each individual was asked to intubate the simulator using their assigned method, which was only disclosed immediately prior to the start of the procedure. We recorded the time from the beginning of laryngoscopy (i.e. skin contact with the laryngoscope) to the end (laryngoscope was removed from the mouth with catheter in place). If students had difficulties to complete intratracheal catheter placement, no help was provided and the clock was stopped only after proper catheter positioning (continuously assessed by the investigators on the video-laryngoscopy screen) and the end of laryngoscopy. Since neonatologists are familiar with both techniques, no prior training was offered in this group.

### In vivo subjects

The second part of this study was conducted between November 1^st^ 2019 and April 30^th^ 2020. Subjects who fulfilled all following inclusion criteria were enrolled. All pregnant women admitted for childbirth with a high risk for preterm delivery were prescreened and their consent to participate in the study was requested prior to birth. Gestational age was estimated based on last menstrual period and corrected by crown-rump-length measurement during first trimester prenatal ultrasound.

Inclusion criteria: (all of the inclusion criteria had to be met).

- Preterm infants born less than 37 weeks of gestation
- Treating physician in charge of admission decides to administer intratracheal surfactant via standardized institution- LISA protocol (regardless of this study) (see Supplement file 1)
- Written informed consent signed by caregivers or legal representative to participate

Exclusion criteria:

- Refusal to participate in study or not providing written informed consent by caregivers/parents
- Treating physician decides to use different route of surfactant administration or does not adhere to LISA protocol.
- Rupture of membranes (ROM) at less than 22 weeks of gestation or more than 6 weeks before birth
- Estimated birth weight < 3rd percentile using 2013 Fenton growth trajectories [19]
- Twins with feto-fetal transfusion syndrome (FFTS) and FFTS being the cause of premature delivery
- Contraindications listed in the LISAcath® or Nasogastric Tube manual (esophageal/pharyngeal varices or other vascular lesions, esophageal/pharyngeal tumor, nasal fracture, skull fracture, known allergy to material)

### Methodology

Patients were randomized to receive surfactant replacement via nasogastric tube or LISAcath® intubation during LISA protocol without pre-medication (supplement file 1) [5, 6]. Demographic, clinical, laboratory and microbiologic data were obtained from electronic patient records. Randomization was done using block randomization (https://www.randomizer.org). At the beginning of each LISA procedure, the treating physician was given a sealed envelope, a randomized method written on the inside.

In method 1 (group 1), we used a flexible feeding tube (Unomedical Charrière 4 ConvaTec UK) that needs to be inserted into the baby’s trachea using Magill forceps to the desired depth. After correct placement of the tube, the forceps and laryngoscope were removed and surfactant was injected. In method 2 (group 2), physicians had to use a straight semi-rigid catheter, LISAcath® (Chiesi Farmaceutici S.p.a. Italy) that is capable of being slightly shaped into a curve to facilitate intubation without the use forceps. Centimeter markers from the tip of the tube serve as index to estimate intubation depth. No pharmacologic procedural sedation and analgesia other than swaddling was used and physical stimulation and a caffeine-citrate loading dose of 20 mg/kg estimated birth weight was used to encourage spontaneous breathing. Laryngoscope Miller 00 blades were used for infants < 1 kg and 00 or 1 blades were used for infants > 1 kg. During neonatal resuscitation, study personal recorded the infant’s vital signs and measured laryngoscopy time using either catheter. The primary endpoint was successful intratracheal tube placement.

### Statistics

In a first step data was analyzed descriptively according to scale level. Boxplots were used for visualization of data. For investigation of differences in ordinal/metric variables Wilcoxon-tests were performed instead of t-tests due to non-normality of data (assessed via q-q-plots). Differences regarding nominal variables were tested with Fisher’s exact test due to small absolute cell frequencies. In addition, odds ratios were calculated as measure of effect. For the primary hypothesis an ANCOVA was performed additionally to Wilcoxon-test to adjust for effect of birth weight on procedure duration.

All results are presented along with 95%-confidence intervals. For outcomes where the median is presented, confidence intervals were calculated by bootstrap-approach. All tests were carried out at 5%-significance level. We opted against *p*-value correction for secondary outcomes due to exploratory style of the analyses. Data has been analyzed using R (Version 1.4.1106). Sample size estimation was mainly dependent on data from the initial study involving medical students, nurses and doctors (excluding students with procedure times > 120 s). All calculations were done for alpha = 0.05 and beta = 0.80. A time difference of 30 s was considered as a relevant effect (Δ), and 1:1 group allocation was assumed. We considered a laryngoscopy duration greater than 30 s as a relevant effect because prolonged (i.e. more than 30 s) laryngoscopy poses significant discomfort for the baby and an increased risk of desaturation or bradycardia. In the literature, the reported mean duration of laryngoscopy is between 24 and 37 s in a difficult neonatal airway model using different catheters [18].

#### Ethics

The study protocol was reviewed and approved by the IRB (ethics commission Salzburg) and all investigators adhered to the declaration of Helsinki and good clinical practice (GCP) (supplementary material). Further, the study adheres to CONSORT guidelines (www.consort-statement.org).

## Results

### Preliminary data

A total of 40 students, 40 nurses and 12 neonatologists were recruited to participate in the study. Table 1 summarizes demographic characteristics, prior experience in neonatal intubation and procedural durations. The majority of the participants (90% students, 65% nurses and 91.7% of doctors preferred the semi-rigid catheter. Mean speed of laryngoscopy in all groups was 35.2 vs 89.1 s (students; *p* < 0.0001), 42 vs 80.1 s (nurses, *p* < 0.0001) and 24.4 vs 39 s (doctors, p.13).

**Table 1:** Demographic data and procedural duration of students nurses and doctors

	**Students**	**Nurses**	**Doctors**
**Sex**^**a**^**:** n (%)			
**female**	21 (52.5%)	35 (87.5%)	5 (41.7%)
**male**	16 (47.5%)	5 (12.5%)	7 (58.3%)
**Level of experience**^**b**^**:** n (%)			
**1** *(1*^*st*^ *year student / nurse or doctor with 1–3 years of experience)*	8 (20%)	9 (22.5%)	4 (33.3%)
**2** *(2*^*nd*^* year student / nurse or doctor with 4–6 years of experience)*	15 (37.5%)	14 (35%)	2 (16.7%)
**3** *(3*^*rd*^* year student / nurse or doctor with 7–10 years of experience)*	5 (12.5%)	5 (12.5%)	2 (16.7%)
**4** *(4*^*th*^* year student / nurse or doctor with* > *10 years of experience)*	12 (30%)	12 (30%)	4 (33.3%)
**Prior intubation experience:** n (%)			
**yes**	18 (45%)	7 (17.5%)	12 (100)
**no**	22 (55%)	33 (82.5%)	0 (0%)
**Preferred method:** n (%)			
**Semi-rigid tube**	36 (90%)	26 (65%)	11 (91.7%)
**NG tube**	4 (10%)	14 (35%)	1 (8.3%)
**Laryngoscopy time**^**c**^ mean (95% CI)			
**Semi-rigid tube**	35.2 (22.1–48.3)	42.0 (26.7–57.5)	24.4 (5.5–43.2)
**NG tube**	89.1 (61.3–117)	80.1 (61.1–99.1)	39 (15.3–62.6)
*p*-value^4^	< 0.0001	< 0.0001	0.1320

^a^ not disclosed n 3 (7,5%)

^b^ expressed as years of medical school (students), neonatal ward experience (nurses) neonatal training (doctors)

c in seconds separated by sex

^4^ Mann–Whitney U

### In vivo* study*

**Fig. 2:**
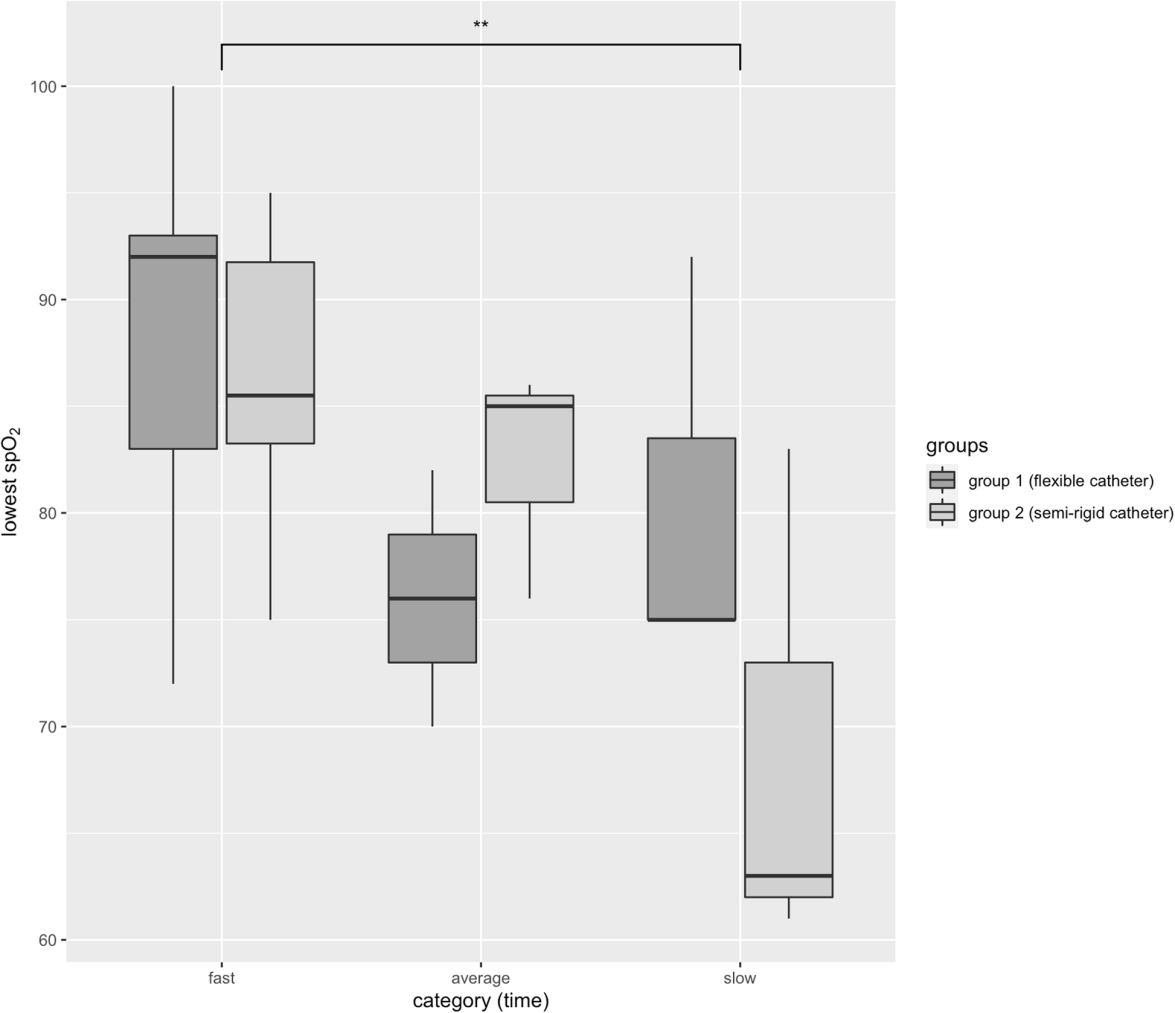
lowest oxygen saturation during laryngoscopy. ** *p* (.0030)

Thirty-two newborns were enrolled in this study, with one later exclusion due to missing consent form. Table 2 summarizes patient demographics. Mean gestational age and birth weight were similar in both groups (1009 g and 27 6/7 weeks in method 1 group; 1128 g and 28 1/7 weeks in method 2 group). During the in vivo study period, a total of 43 laryngoscopies (including repeated attempts), performed only by neonatologists with more than three years of professional experience, were recorded. All patients were deemed to be “stable” prior to laryngoscopy (i.e. heart-rate > 100 bpm, peripheral oxygen saturation > 85% at any FiO2 and spontaneous breathing) according to the institution LISA protocol (supplement file 1) in the first 10 min of life. In 11 (25.6%) cases laryngoscopy failed during the first attempt but correct tube placement was achieved during a second attempt. A higher number of failed attempts (43.8% vs. 26.7%) occurred in group 1 than when using a semi-rigid device but there was no significant difference between both groups on first attempt (OR 2.15, p.46). In one case (2.3%), laryngoscopy had to be performed thrice. In another case, the physician changed from group 1 to group 2 after unsuccessful catherization. In this case, only the first laryngoscopy is counted as an unsuccessful event since the second attempt was not performed as part of the orginially assigned group. Table 3 summarizes procedure durations, heart rate and oxygen saturation during laryngoscopy. In order to exclude outliers, data that included 80% of fastest attempts is presented separately (thus laryngoscopy attempts longer than 100 s were excluded. This was done because a laryngoscopy attempt of more than 100 s is likely to be influenced by various other issues; e.g.repositioning of the baby during laryngoscopy, monitoring of vital signs, increasing oxygen delivery, vigorous suctioning, second catheter placement attempt without removing the laryngoscope, etc.). Three subgroups (fast, average, slow), were defined by laryngoscopy durations of less than 54 s; 55 to 83 s and 84 to 136 s (intervals created as thirds of the difference between maximum and minimum duration; See below for discussion of time intervals). Figure 2 shows lowest peripheral oxygen saturations between each group and subgroup. There was no significant difference between the fast and average paced group (p.26), with respect to their lowest peripheral oxygen saturation, but a significant difference was found between the fast and the slow group (p.0030). In addition, birth weight or gestational age showed no notable statistical impact on procedure duration with no significance (ANCOVA p.70 and p.95 respectively). When vital signs were analyzed, no bradycardia less than 70 bpm was recorded, but 4 (26.7% patients in group 2 experienced heart rates less than 100 beats per minute; bpm). No newborn showed any sign of laryngeal injury or soft tissue tear within the first week following laryngoscopy. Intraventricular hemorrhage (IVH) other than IVH grade 1 occurred in 4 (12.9%) patients, two from each group. Diagnosis of IVH was made on 1^st^ day of life in two subjects (group 1 and 2), on the 4^th^ day in one patient (group 2) and the 5^th^ day in another subject (group 1). One patient died on the 9^th^ day of life due to complications associated with a necrotizing enterocolitis.

**Table 2:** Patient demographics

	**Group 1**	**Group 2**
**Gestational age**^**a**^		
Mean (95% CI)	27 6/7 (26 5/7—29 0/7)	28 1/7 (26 5/7—29 4/7)
23 + 0/7 to 25 + 6/7	*N* = 4 (12.9%)	*N* = 3 (9.7%)
26 + 0/7 to 28 + 6/7	*N* = 8 (25.8%)	*N* = 5 (12.1%)
29 + 0/7 to 32 + 0/7 = 1	*N* = 4 (12.9%)	*N* = 7 (22.6%)
**Birth weight**		
Mean (95% CI)	1009 (932–1288)	1128 (892–1202)
500 to 999 g	*N* = 8 (25.8%)	*N* = 7 (22.6%)
1000 to 1499 g	*N* = 7 (22.6%)	*N* = 5 (12.1%)
1500 to 2000 g	*N* = 1 (3.2%)	*N* = 3 (9.7%)
**Total number of participants**	*N* = 16 (51.7%)	*N* = 15 (48.4%)

Group 1: nasogastric feeding tube inserted with Magill forceps

Group 2: semi-rigid tube

^a^ Gestational age according to prenatal ultrasound

**Table 3:** In vivo results; Group 1: flexible nasogastric tube, group 2: semi-rigid catheter

	**Group 1**	**Group 2**	Statistics
**Number of infants** randomized to group (prior to first attempt) n (%)	16 (51.6%)	15 (48.4%)	
**Laryngoscopy attempts**	23	20	
**Failed first attempt** n (%)	7 (43.8%)^b^	4 (26.7%)^b^	Failed first attempt OR 2.15 Fisher’s exact *p* = .46
**Procedure duration** (seconds)^a^			
Mean	61.07 (44.9)^a^	64.87 (50.7)^a^	Wilcoxon: *p* = .771 (.623^a^)
95% CI	40.6–81.6 (32–58)^a^	42.4–79.7 (37.6–63.8)^a^	
Median	49 (43.5)^a^	49 (44)^a^	
Min	13 (13)^a^	27 (27)^a^	
Max	136 (83)^a^	130 (96)^a^	
**peripheral O2 Saturation** % (median, 95% CI)^a^			
initial	95 (94)^a^ [(91–98) (89.5–100)^a^]	92 (91.5)^a^ [(90–97) (88–96)^a^]	Wilcoxon: *p* = .153 (.446)^a^
lowest	91 (92)^a^ [(90–100) (88–100)^a^]	85 (85)^a^ [(84–94) (81.5–89.5)^a^]	Wilcoxon: *p* = .299 (.39)^a^
**heart rate** (median, 95% CI)^a^			
initial	163 (156)^a^ [(150–183) (137–171)^a^]	159 (157)^a^ [(148–161) (137–159)^a^]	Wilcoxon *p* = .662 (.432)^a^
lowest	141 (142)^a^ [(115–150) (111–151)^a^]	135 (135)^a^ [(115–142) (113–143)^a^]	Wilcoxon *p* = .459 (.459)^a^

^a^ brackets show data with outlier excluded (I.e. 20% of the longest laryngoscopies excluded)

^b^ one infant was changed from group 1 to group 2 after the first failed attempt and the doctor therefore performed a second attempt using a semi-rigid catheter as prescribed for group 2. Failed laryngoscopy total attempts group 1 (including infant which was transferred to group 2): 8 (34.8%); group 2: 4 (20%)

## Discussion

First described in 1992, the use of small catheters for surfactant application in order to avoid endotracheal intubation and positive pressure ventilation, is now the primary recommended method in early postnatal surfactant application to treat RDS [5, 8, 20].

In alignment with current recommendations to use minimally invasive approaches during surfactant administration in preterm infants and ongoing, high-quality clinical trials, this study aimed to evaluate the clinical impact of semi-rigid versus flexible catheters, for laryngoscopy and surfactant administration [6, 10, 11, 14].

### In-vitro differences between flexible and semi-rigid catheters

Currently, there exist no high-quality real-live in-vivo data on the application of different catheters during LISA. In 2017, Rigo and colleagues already published in vitro data asserting that intratracheal catheter placement is both faster and easier when using a rigid device and that the LISAcath® was found to be the preferred semi-rigid catheter by neonatologists during experimentation on airway dummies [18]. In our study, using a more sophisticated airway model to simulate anatomic conditions in extremely premature infants (< 28 weeks of gestation) we found similar results in students, nurses and doctors as found by Rigo. Laryngoscopy and soft flexible catheter handling with Magill forceps is thought to be especially challenging if there is no prior nasotracheal intubation experience [6]. However, with a higher level of medical expertise and neonatal intensive care training, the time difference of laryngoscopy became negligible and we did not find any difference in laryngoscopy duration in doctors with profound intubation experience. In a pan European survey (> 300 neonatologists, and 37 countries) the majority of neonatologists (56%) use standard flexible infant feeding tubes during LISA procedures, 34% use vascular catheters and 15% suction catheters. The size ranged from 2.5 to 5 French and two thirds use Magill forceps to insert the catheter [21]. Despite widespread use of flexible catheters inserted with forceps, when tested in vitro against semi-rigid catheters, the overwhelming majority of participants favored the latter, which could be introduced faster and without the use of additional devices [5, 17, 18]. The vast majority of nurses in our study were female. However, since there were similar numbers of female and male doctors and students and we did not find a sex difference, we consider this issue irrelevant.

### Clinical implications of laryngoscopy time and catheter properties

Currently, no in vivo data exist on tracheal catheterization speed using different catheters. In our study of 31 premature infants, laryngoscopy failed or was interrupted 12 (27.9%) times (in 11 cases the first attempt was interrupted), but there was no significant difference in the number of failed attempts between the groups. In literature, the average rate of failed laryngoscopy on the first attempt was reported to be 10–48% using a sample with similar demographics of premature infants [7, 13, 22]. Laryngoscopy and intrapharyngeal instrument handling are major causes of discomfort and pain during surfactant application [23]. However, most randomized controlled trials do not use a standardized premedication before LISA / MIST procedures [6, 24]. Especially when opting to avoid the use of pharmacological and potentially respiration compromising sedatives it is crucial to use a fast and straightforward method to minimize laryngoscopy time [18, 24]. Early and correct tube placement equals less pain and discomfort.

Additionally, laryngoscopy causes several deleterious physiologic responses including hypoxemia, bradycardia and intracranial hypertension. Hypoxemia itself is linked to prolonged laryngoscopy and subsequent airway obstruction [25]. Bradycardia during intubation is mostly caused by parasympathetic stimulation of catheter and laryngoscope and baroreceptor response to increased blood pressure [25]. This is a reason why some neonatology units premedicate infants with atropine in order to avoid dangerous drops in heart rate during LISA/MIST [26, 27]. As in previous studies, in order to reduce the risk of post-procedural respiratory and cardiovascular depression, our protocol does not make use of medical sedatives, analgetics or narcotics and therefore, efficiency is imperative to minimizing patient discomfort and additional clinical risk factors [17, 18, 26, 28]. In our patient collective, the majority of laryngoscopies could be performed in under 60 s with a low rate of failure which signifies the staff’s high level of LISA and intubation proficiency. We did not find a significant difference in speed using one catheter over the other when intubation was performed by experienced neonatologists. Further, no significant difference has been found when analyses are adjusted for birthweight which we consider the most important anthropomorphic factor impacting laryngoscopy compared to gestational age, sex or body length. Figure 2 does however emphasize that longer durations of laryngoscopy were associated with significant higher drops in peripheral oxygen saturation. Our data shows no difference in occurrence of low bradycardias (< 70 bpm) in either group, but heart rates of less than 100 bpm were exclusively found in group 2. In these cases, the semi-rigid catheter may have caused more intense vagal stimulation during laryngeal insertion than the softer feeding tube even when inserted with Magill forceps. Randomized controlled trials using LISA protocols similar to ours without the preliminary use of narcotic, sedative or vasoactive drugs, observed similar results as our study [7, 13, 15]. These studies report that all infants remained stable, without significant bradycardias and desaturations when LISA was performed with either flexible feeding tubes or semi-rigid vascular catheters [7, 13, 15]. However, these studies reported only physiological parameters during surfactant application (laryngoscopy and drug instillation) and did not compare procedure properties when using flexible and semi-rigid catheters. Notably, no infant experienced soft tissue lacerations or airway injury following laryngoscopy. Two patients from each group experienced IVH on the first day of life. A known possible complication, besides prematurity itself, increased intracranial pressure during and after laryngoscopy poses a risk for intraventricular hemorrhage [29]. In our case, MRI and sonography results deemed hemorrhaging not to be associated with treatment and likely originating prior to birth.

The vast majority of institutions who perform LISA procedures use catheters that lack medical device licensing and CE labeling for this particular application [21]. Besides forceps, newly developed catheter mounts have been tried to facilitate tracheal insertion of flexible tubes, but clinical in vivo data is still missing and devices are solely tested during research studies and not readily available [4]. LISAcath® is currently one of the few catheters, ISO licensed to be used in preterm infants for surfactant administration. Especially in institutions with less experienced staff, its ease of use, and overall positive feedback by participants and official licensing makes it a valid choice.

Airway simulator and premature infant mannequins do not reflect NICU real-live resuscitation circumstances. Even though we used a highly sophisticated airway model in the first part of the study, laryngoscopy time differences might be different in real-live. Further, the single center design and a small sample size are major limitations of this study. Smaller differences in laryngoscopy times may be undetected due to the small sample size. There is no published or established definition of fast versus slow laryngoscopy. Our definition of procedure speed was calculated by dividing the difference of our fastest and slowest recorded laryngoscopy completion times into thirds. However, choosing a different group calculation, would yield different results. In addition, since standardized operating procedures may vary between institutions, our data predominantly reflects our department. Despite this, the fact that our department has adhered to the same protocol for preterm resuscitation since 2007, doctors and nurses have been well trained and therefore laryngoscopy speed alterations are likely to reflect true time differences. Further, this study does not account for personal preferences of the doctors performing the procedure which may impact their performance. However, because all study members use both catheter devices in day to day clinical work and randomization was done immediately before neonatal resuscitation, this effect is minimized.

## Conclusion

This is the first in vivo study to investigate laryngoscopy speed in premature infants during minimal invasive surfactant application using two different catheter systems. Even though, both neonatologists and inexperienced users (nurses and students) overwhelmingly preferred the use of semi-rigid catheters, we did not find a significant difference in laryngoscopy duration when compared to flexible nasogastric tubes inserted with Magill forceps in vivo and in vitro. Data suggests that especially in the hands of highly experienced intubation experts, duration of laryngoscopy is similar in both groups and clinical benefits other than procedure speed need to be sought out in further studies. Due to the small sample size, minor differences may have been undetected, but our results suggest that a flexible feeding tube can easily be used for minimal invasive surfactant application, with similar complication rates but lower rates of bradycardia than semi-rigid devices.

## Supplementary Information

**Additional file 1.**

## Data Availability

All data generated or analyzed during this study are included in this article [and/or] its supplementary material files. Further enquiries can be directed to the corresponding author.

## Abbreviations

BPD: Broncho pulmonary dysplasia
BPM: Beats per minute
CPAP: Continuous positive airway pressure
ELBW: Extremely low birth weight infant
FFTS: Feto-fetal transfusion syndrome
FiO2: Fraction of inspired oxygen
GCP: Good clinical practice
IVH: Intraventricular hemorrhage
LISA: Less invasive surfactant application
MIST: Minimal invasive surfactant treatment
NICU: Neonatal intensive care unit
RDS: Respiratory distress syndrome
ROM: Rupture of membranes
