# Supplementary material for: A pilot study of evaluation of semi-rigid and flexible catheters for less invasive surfactant administration in preterm infants with respiratory distress syndrome—a randomized controlled trial
Source: BMC Pediatr. 2022 Nov 4;22:637. doi: 10.1186/s12887-022-03714-3 (PMC9635199; doi:10.1186/s12887-022-03714-3)
Supplement: Supplementary file 1 — Additional file 1. [file 12887_2022_3714_MOESM1_ESM.doc]

# Evaluation Of Rigid And Flexible Catheters For Less Invasive Surfactant Administration In Preterm Infants With Respiratory Distress Syndrome

#

**Principal Investigator:**

**Assoc. Prof. Dr. Martin Wald**

**University Hospital Salzburg**

**Dept. of Pediatrics and Adolescent Medicine**

**Division of Neonatology**

**Müllner-Haupt-Straße 48**

**A-5020 Salzburg**

**Mail:** [**M.Wald@salk.at**](mailto:M.Wald@salk.at)

**Tel: +43(0)57255-26125**

# Collaborating institutions

| **Investigator** | **Institution** |
| --- | --- |
| Anna Eichhorn | University Hospital of Salzburg  Dept. of Pediatrics and Adolescent Medicine  Division of Neonatology |
| Dr. Lorenz Auer-Hackenberg | University Hospital of Salzburg  Dept. of Pediatrics and Adolescent Medicine  Division of Neonatology |
| Dr. Johannes Brandner | University Hospital of Salzburg  Dept. of Pediatrics and Adolescent Medicine  Division of Neonatology |
| Dr. Christof Weisser | University Hospital of Salzburg  Dept. of Pediatrics and Adolescent Medicine  Division of Neonatology |

**Table of content**

Evaluation Of Rigid And Flexible Catheters For Less Invasive Surfactant Administration In Preterm Infants With Respiratory Distress Syndrome [1](#__RefHeading___Toc13247164)

Collaborating institutions [2](#__RefHeading___Toc13247165)

List of abbreviations [4](#__RefHeading___Toc13247166)

Abstract (Deutsche Zusammenfassung) [5](#__RefHeading___Toc13247167)

Goals and Objectives: [5](#__RefHeading___Toc13247168)

Background [6](#__RefHeading___Toc13247169)

Objectives and purpose [8](#__RefHeading___Toc13247170)

Hypotheses [8](#__RefHeading___Toc13247171)

1. Time needed for successful endotracheal catheterisation [8](#__RefHeading___Toc13247172)

2. Less laryngoscopy associated complications during surfactant application using LISA protocol [8](#__RefHeading___Toc13247173)

Study methodology [9](#__RefHeading___Toc13247174)

Preleminary study: Simulation of surfactant application in preterm infants at 27 weeks of gestation. [9](#__RefHeading___Toc13247175)

In vivo study: [10](#__RefHeading___Toc13247176)

Study design [10](#__RefHeading___Toc13247177)

Study population [10](#__RefHeading___Toc13247178)

Study site [11](#__RefHeading___Toc13247179)

Sample size [14](#__RefHeading___Toc13247180)

1. Data acquisition [16](#__RefHeading___Toc13247181)

a. Time of laryngoscopy [16](#__RefHeading___Toc13247182)

b. Vital signs [16](#__RefHeading___Toc13247183)

Data management [16](#__RefHeading___Toc13247184)

Analysis/Statistics [17](#__RefHeading___Toc13247185)

Ethical considerations [18](#__RefHeading___Toc13247186)

Eingehaltene Normen [18](#__RefHeading___Toc13247187)

References [21](#__RefHeading___Toc13247188)

Appendices [23](#__RefHeading___Toc13247189)

1. Student questionnaire [23](#__RefHeading___Toc13247190)

2. LISA SOP (german) [24](#__RefHeading___Toc13247191)

3. Case-Report-Form [28](#__RefHeading___Toc13247192)

# List of abbreviations

| BPD | broncho pulmonary dysplasia |
| --- | --- |
| CPAP | continuous positive airway pressure |
| ELBW | extremely low birth weight infant |
| FFTS | feto-fetal transfusion syndrome |
| FiO2 | fraction of inspired oxygen |
| GCP | good clinical practice |
| HR | heart rate |
| NICU | neonatal intensive care unit |
| LISA | less Invasive Surfactant Application |
| RDS | respiratory distress syndrome |
| ROM | rupture of membranes |
| SAE | severe adverse event |
| SOP | standard operating procedure |
| SpO2 | oxygen saturation (transcutaneous) % |

# Abstract (Deutsche Zusammenfassung)

Bei extremen Frühgeborenen ist häufig eine exogene Surfactant Gabe im Rahmen der Erstversorgung nach Geburt notwendig zur Verhinderung bzw. Behandlung des Atemnotsyndroms. Derzeit existieren unterschiedliche Empfehlungen zur optimalen intratrachealen Applikationsmethode. International akkreditiert und seit 2012 an unserer Abteilung praktiziert ist eine minimal invasive Form (LISA Verfahren: less invasive surfactant administration) bei der eine Nasogastral-Sonde verwendet wird, die mittels Magill Zange in die Trachea eingeführt wird. Seit einiger Zeit steht nun ein weiterer Katheter zur Verfügung, der wegen der deutlich kürzeren Länge und Steifigkeit ohne Hilfsmittel händisch in die Trachea eingebracht werden kann.

Das Ziel dieser klinischen Medizinprodukteprüfung ist der Vergleich beider Sonden im Rahmen des LISA Protokolls an frühgeborenen Kindern mit Atemnotsyndrom. Im Detail werden dabei die Laryngoskopiedauer beider Methoden und die damit verbundenen Auswirkungen auf die kindlichen Vitalparameter untersucht. Die Studie läuft von 01. November 2019 bis zum 30. April 2020. In diesem Zeitraum sollen Kinder die unmittelbar nach der Geburt im Rahmen eines Atemnotsyndroms bzw. auf Grund der extremen Frühgeburtlichkeit Surfactant mittels LISA Protokoll erhalten eingeschlossen werden. Eingeschlossene Kinder werden mittels Blockrandomisierung einem der beiden Kathetersysteme zugeordnet welches in weiterer Folge bei einer Surfactant Applikation nach LISA Schema zur Verwendung kommen soll.

# Goals and Objectives:

The overall aim of this study is to determine the differences between two surfactant administration catheters in preterm infants.

| **MAIN OBJECTIVES OF THIS STUDY PROTOCOL**   1. Compare laryngoscopy times of LISAcath® endotracheal catheters with conventional nasogastric tube for intratracheal surfactant application. |
| --- |

The main objective of the study is to determine the time needed for successful endotracheal catheterisation to deliver surfactant in preterm infants.

# Background

In neonatology, the administration of exogenous surfactant into the lungs of premature newborns with respiratory distress syndrome is a well-recognized and important tool to increase survival^1^ especially in extremely low birth weight (ELBW) infants and decrease neurologic morbidity (such as intracranial haemorrhage), broncho-pulmonary dysplasia (BPD) and other chronic lung disease^2^. Today, surfactant is one of the cornerstones in the therapy of respiratory distress syndrome (RDS) in newborns, its application is already extensively studied and there exist a variety of modalities of surfactant delivery methods^3^.

Minimal invasive methods for surfactant application.

The application of continuous positive airway pressure (CPAP) together with the application of CPAP immediately after delivery is another important factor during resuscitation of extremely preterm babies. Studies showed that RDS is an important risk factor for CPAP failure which led the way to surfactant application in the delivery room^4^. The optimal mode and time of surfactant delivery into the respiratory tract is matter of different investigations. In our center we started to implement a standardized approach in premature newborn resuscitation immediately after delivery, based on the principle of minimal invasive surfactant application^5^. Babies born at a gestational age less than 28 weeks or premature infants who develop signs of respiratory distress (e.g. oxygen requirement with an FiO2 of .3 or greater after birth) receive CPAP breathing support immediately after birth (see Appendix 2). After successful cardiorespiratory adaptation, intravenous access is obtained and a loading dose of caffeine citrate is injected. While remaining on pharyngeal CPAP, after caffeine administration, neonatologists will start laryngoscopy and after intratracheal placement of a small tube (e.g. Ch 4 feeding tube; see below) inject surfactant into the baby’s lungs. This technique, labeled the Less Invasive Surfactant Application procedure (LISA), was started in 2012 at our center. Every staff member (nurses, neonatologists, etc.) is well trained in the particular sequence of steps in the LISA protocol, thus ensuring standardized best practice in every circumstance. During the LISA procedure, babies do not need to be intubated or sedated for surfactant application and the early application of CPAP supports lung recruitment and spontaneous breathing. After all this particular technique reduces stress and secondary complications of mechanical ventilation or intubation^6^.

Despite wide acceptance of LISA as a standardized protocol for surfactant application and its adoption of use in many centers worldwide, the optimal catheter to use for intubation and surfactant administration remains of debate. Preliminary data already underscores the advantages of rigid catheters compared to flexible feeding tubes^7–9^. Most recently, Rigo and colleagues showed that the use of stylet-guided catheters or rigid tubes is significantly faster than the use of flexible feeding tubes (inserted with the help of Magill forceps) during intubation of neonatal airway dummies.

In our center we currently use flexible feeding tubes as the main method during LISA. Despite usage without CE certification the use of feeding tube is currently the international gold-standard in minimal invasive surfactant application according to LISA protocol.^5^ In addition, since 2017 we were further able to use shorter rigid catheters for intubation. Because of initial very positive feedback by our neonatologists, the department plans to change their LISA protocol as soon as the rigid catheter for surfactant application becomes commercially available. This study aims to investigate the advantages of either technique. ^5, 7-9^

Preliminary data

As part of this study we wanted to compare the average time needed for laryngoscopy using both techniques in untrained medical students. In February 2019, students from the first four years of medical school (Paracelsus Medical University Salzburg) without particular knowledge or experience in neonatology or intubation were asked to participate. Compared to Rigo and colleagues we used a more sophisticated airway model that better simulates anatomic conditions in extremely premature infants (<28 weeks of gestation).

# Objectives and purpose

The overall perspective of this study is to improve our knowledge on different surfactant application methods. Especially in the time of less invasive surfactant application approaches it is crucial to determine the best mode of surfactant delivery.

# Hypotheses

The following underlying hypotheses are driving the respective study. Study design and endpoints have been chosen to adequately address these research questions:

### Time needed for successful endotracheal catheterisation

- Using the rigid LISAcath® system makes it easier and faster to successfully intubate a preterm infant for surfactant application.

### Less laryngoscopy associated complications during surfactant application using LISA protocol

- Laryngoscopy associated complications (i.e. oropharyngeal bleeding, desaturation <60% during procedure, intracranial bleeding and oropharyngeal soft tissue injury during and after surfactant application in preterm infants on non-invasive ventilator support and respiratory distress syndrome (RDS)

# Study methodology

The description of the methodology of the study is depicted below. Most importantly, all participants of this study will be managed following routine patient care at the respective department and the decision to participate in this cohort study will therefore not adversely affect patient care. However, data will be extracted from routine medical charts and recorded during routine care of preterm infants. Therefore, informed consent is required from caregiver’s or legal representatives (mainly parents) before participation in the study protocol (see below). As to date, both techniques are well established methods for endotracheal surfactant delivery in preterm infants and at our center, all doctors in charge of patient admission are well trained in both techniques tested. Currently both catheter systems are used in routine patient care.

### Preleminary study: Simulation of surfactant application in preterm infants at 27 weeks of gestation.

In the primary part of the study (February 2019) the goal was to gather preliminary data on the feasibility and fastness of surfactant application using either LISAcath® or conventional nasogastric tube on untrained medical students.

At the Paracelsus Medical University we contacted medical students from the first four years of medical school to participate in the study. 41 participants were grouped to groups of four to six students. After 10 minutes of basic introduction, both techniques were demonstrated using a video laryngoscope and the preterm baby simulation dummy “Paul” (SIMCharacters GmbH, Vienna). “Paul” is a state-of-the-art preterm baby simulator representing an infant at a weight of 1000g and 27+3/7 weeks of gestation. Currently it represents the most realistic preterm infant airway model in neonatology. After the introduction all participants had to successfully intubate the dummy using both techniques. During training, all students were supervised and coached by the investigators. After 50 minutes, students had to leave the simulation room. We used a block-randomisation of 4 to randomise students to either technique. They had to fill out a questionnaire assessing basic demographic data and previous intubation attempts and trainings. Students were then one-by-one asked to intubate the simulator after disclosing their assigned method. We recorded the time from the beginning of laryngoscopy (i.e. skin contact with the laryngoscope) to the end (laryngoscope was removed from the mouth and catheter in place). If students had difficulties to complete intratracheal catheter placement, no help was provided and the clock was stopped only after proper catheter positioning (continuously assed by the investigators on the video-laryngoscopy screen) and the end of laryngoscopy. See supplement 1: Students questionnaire.

Results are shown in Table 1

Table 1: Preliminary Data

| Subject # | Previous Intubation (Nein = no; Ja=yes) | preferred method  *(Magensonde = nasogastric tube)* | assigned method *(Magensonde= nasogastric tube)* | handedness *(r=right; l= left)* | Gender *(w = female; m= male)* | Age (yrs) | Medical school year | Time needed for intubation |
| --- | --- | --- | --- | --- | --- | --- | --- | --- |
| 1 | Nein | LISACath | Magensonde | r | w | 18-20 | 2 | 43,98 |
| 2 | Nein | LISACath | Magensonde | r | w | 18-20 | 2 | 01:19,2 |
| 3 | Nein | LISACath | LISACath | r | m | 18-20 | 1 | 40,79 |
| 4 | Nein | LISACath | LISACath | r | m | 18-20 | 2 | 19,12 |
| 5 | Nein | LISACath | Magensonde | r | m | 20-22 | 2 | 33,37 |
| 6 | Nein | LISACath | Magensonde | r | w | 18-20 | 2 | 01:01,6 |
| 7 | Nein | LISACath | LISACath | l | w | 20-22 | 2 | 22,03 |
| 8 | Nein | LISACath | LISACath | r | w | 25 < | 2 | 21,61 |
| 9 | Nein | LISACath | Magensonde | l | m | 22-25 | 2 | 02:17,2 |
| 10 | Nein | - | Magensonde | l | m | 20-22 | 2 | 05:07,5 |
| 11 | Ja | LISACath | LISACath | r | m | 25 < | 3 | 19,28 |
| 12 | Nein | LISACath | LISACath | r | m | 25 < | 3 | 52,59 |
| 13 | Ja | LISACath | Magensonde | r | w | 20-22 | 4 | 01:51,1 |
| 14 | Ja | Magensonde | Magensonde | r | w | 22-25 | 4 | 02:21,7 |
| 15 | Nein | LISACath | LISACath | r | w | 20-22 | 4 | 24,53 |
| 16 | Ja | LISACath | LISACath | r | w | 20-22 | 2 | 32,02 |
| 17 | Ja | LISACath | LISACath | r | w | 22 | 4 | 28,32 |
| 18 | Ja | LISACath | Magensonde | l | m | 25 < | NA | 01:04,2 |
| 19 | Ja | LISACath | LISACath | r | w | 20-22 | 4 | 51,79 |
| 20 | Ja | LISACath | Magensonde | r | w | 20-22 | 3 | 01:19,4 |
| 21 | Ja | LISACath | Magensonde | r | m | 22-25 | 4 | 01:05,2 |
| 22 | Nein | Magensonde | LISACath | r | m | 20-22 | 2 | 02:17,3 |
| 23 | Ja | LISACath | LISACath | r | m | 22,25 | 4 | 21,26 |
| 24 | Ja | LISACath | Magensonde | r | m | 20-22 | 1 | 01:04,1 |
| 25 | Ja | LISACath | LISACath | r | m | 22-25 | 1 | 16,39 |
| 26 | Ja | LISACath | Magensonde | r | m | 22-25 | 1 | 38,61 |
| 27 | Nein | LISACath | LISACath | r | w | 20-22 | 2 | 36,47 |
| 28 | Nein | LISACath | Magensonde | r | w | 20-22 | 2 | 01:59,2 |
| 29 | Nein | LISACath | LISACath | r | w | 18-20 | 1 | 47,93 |
| 30 | Ja | Magensonde | Magensonde | r | m | 22-25 | 4 | 40,89 |
| 31 | Ja | LISACath | Magensonde | r | w | 20-22 | 4 | 37,58 |
| 32 | Nein | Magensonde | Magensonde | l | w | 22-25 | 1 | 01:40,0 |
| 33 | Ja | LISACath | LISACath | r | m | 22-25 | 4 | 23,45 |
| 34 | Ja | LISACath | Magensonde | r | w | 22-25 | 4 | 01:28,6 |
| 35 | Ja | LISACath | LISACath | r | m | 20-22 | 4 | 24,97 |
| 36 | Nein | LISACath | LISACath | r | m | 18-20 | 2 | 19,72 |
| 37 | Nein | LISACath | Magensonde | r | m | 20-22 | 2 | 2;12,26 |
| 38 | Nein | LISACath | Magensonde | r | w | 20 | 3 | 01:32,1 |
| 39 | Nein | LISACath | LISACath | r | w | 18-20 | 1 | 29,34 |
| 40 | Nein | LISACath | Magensonde | r | w | 20-22 | 3 | 33,69 |

## In vivo study:

The second phase of the study will assess the above goals in-vivo.

## Study design

The study will be conducted as an open-label randomized controlled trial.

## Study population

Preterm infants admitted to our department who require surfactant administration using LISA protocol.

Inclusion criteria: (all of the inclusion criteria have to be met)

- Preterm infants born less than 37 weeks of gestation
- Treating physician in charge of admission decides to administer intratracheal surfactant via standardised institution- LISAprotocol (regardless of this study)
- Written informed consent signed by caregivers or legal representative to participate

Exclusion criteria:

1. Refusal to participate in study or not providing written informed consent by caregivers/parents
2. Treating physician decides to use different route of surfactant administration or does not adher to LISA protocol.
3. Rupture of membranes (ROM) at less than 22 weeks of gestation or more than 6 weeks before birth
4. Estimated birth weight < 3^rd^ percentile using 2013 Fenton growth trajectories^10^
5. Twins with feto-fetal transfusion syndrome (FFTS) and FFTS being the cause of premature delivery
6. Contraindications listed in the LISAcath® or Nasogastric Tube manual
   1. esophageal/phayngeal varices or other vascular lesions
   2. esopagheal/pharyngeal tumor
   3. nasal fracture
   4. skull fracture
   5. known allergy to material

Informed consent:

If a patient is thought to meet the inclusion criteria, the investigator physician obtains thorough informed consent prior to enrolment of the patient. The study objectives, protocol and database parameters are explained in full detail according to Good Clinical Practice standards. Informed consent documentation has to be signed (or thumb-printed if the patient is an illiterate) before enrolment and data acquisition. If the person is a minor or otherwise legally incompetent according to Austrian law, a proxy or legal guardian will have to sign informed consent forms (for adults). In case the participant is unable to provide informed consent because his medical condition renders him or her temporarily incompetent, informed consent will be sought as soon as possible. Enrolment of patients without informed consent is not possible. If the patient decides to opt out of the study or does not provide written consent, no further data will be collected. All data gathered up until the time of revocation can still be used for evaluation in this clinical trial. Physicians, who enrol a patient, will provide him or her with written information brochures concerning the study as well as contact information of the investigator physician. If a patient chose to withdraw from the study or withholds consent, he or she will continue to be offered regular patient care.

## Study site

The study will be conducted entirely at the Private Medical University of Salzburg, Landeskrankenhaus Salzburg, Austria. Patients will be recruited at the department of obstetrics and gynaecology. Patients presenting to the obstetric/gynaecology department, and the admitting physicians considers premature birth possible, will be contacted by neonatologists on call and the investigator to provide further information about routine patient care and the study.

#### Methodology:

All patients fulfilling the inclusion criteria will be included in this study.

Between November 1^st^ 2019 and April 30^th^ 2020 patients can be enrolled. If all inclusion criteria are met, patients receive surfactant administration via nasogastric tube or LISAcath® during LISA algorithm. Patients will be randomized to either technique (see below). As part of our routine care, vital parameters (heart rate, oxygen saturation, temperature) are recorded continuously after birth and during the procedure. Investigators on site will be contacted prior to birth and attend the entire LISA procedure. They will record all data needed and oversee randomized allocation to either nasogastric tube or LISAcath® by giving out an envelope with the patient’s identification number on it and the assigned method inside. The doctors responsible will not have access to randomization information (including group allocation or randomization technique) prior to actual randomization. Demographic, clinical, laboratory and microbiologic data will be recorded from electronic patient records. Besides allocating participants to a certain catheter, the study protocol does not alter the type or the course of treatment in a child. If the treating physician deviates from the allocated method at any time, the patient will be excluded from the trial.

LISA procedure SOP:

The department currently executes a certain LISA protocol for every baby receiving surfactant for significant RDS due to prematurity (see appendix 2). Even though the SOP is designed for premature babies born at less than 28 weeks of gestation, the study site currently adheres to this protocol also in later gestational weeks.

Randomization:

Participants will be randomized to either catheter prior to the study. Because of the small sample size, we use a block randomization.^13^ On July 1^st^, after initiation of this study, we use an internet based randomization tool ([https://www.randomizer.org](https://www.randomizer.org/)) to generate random numbers for a blocked design. To achieve an allocation of 50% to each catheter we enter 8 sets of 4 unique, unsorted numbers with a range from 1 to 4 (representing 1 and 3 nasogastric tube and 2 and 4 LISAcath® catheter). Therefore, all participants will be randomized using 6 blocks (AABB, ABAB, ABBA, BBAA, BABA, BAAB) and the system generates a list allocating a certain technique to 32 participants. 32 envelopes marked with numbers 1-32 are then matched with the allocated method from the randomization tool and stored by research personal not involved in the actual LISA procedure. After enrollment, every participant receives a chronological identification number (1-32). At the beginning of each LISA procedure the treating physician will be given the envelope with the patient’s identification number on it and the matched method written on the inside.

Surfactant administration via nasogastric tube:

Currently the standard of care is intratracheal surfactant administration via nasogastric tube (see figure 1).
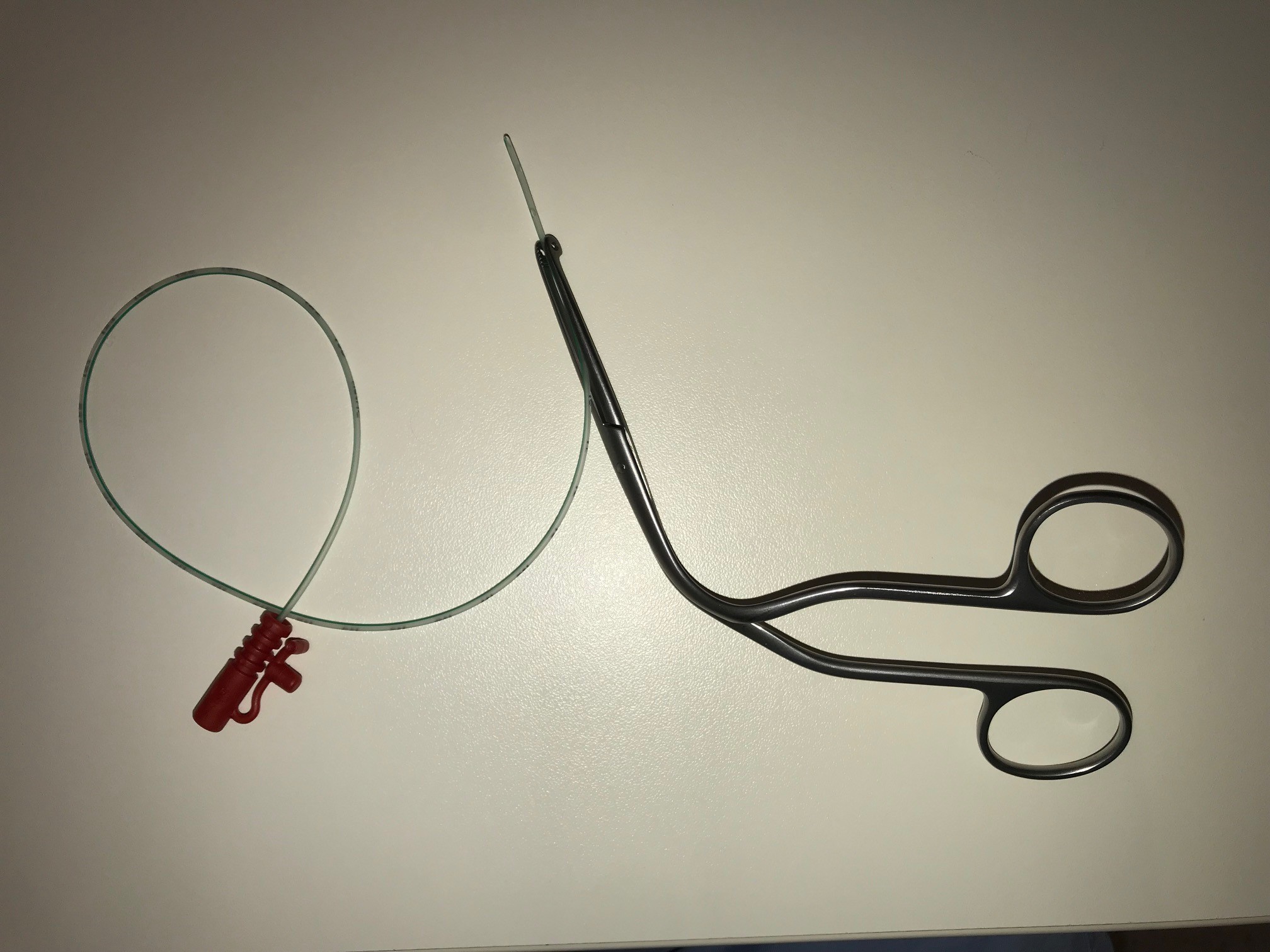


Figure 1. Nasogastric tube (*Unomedical Charrière 4; outer diameter 1.3mm; inner diameter .66mm; length 375mm flexible REF 12021182, ConvaTec UK)* and magill forceps (Photo taken by Lorenz Auer-Hackenberg)

The flexible tube (Ch 4) will be inserted with magill forceps after the desired length from the tip of the tube is highlighted with black permanent marker. After correct placement of the tube, the forceps and the laryngoscope are pulled out of the child and surfactant is injected.

Surfactant administration via LISAcath® system.

A newer option for intratracheal surfactant injection is the LISAcath® system (Figure 2).


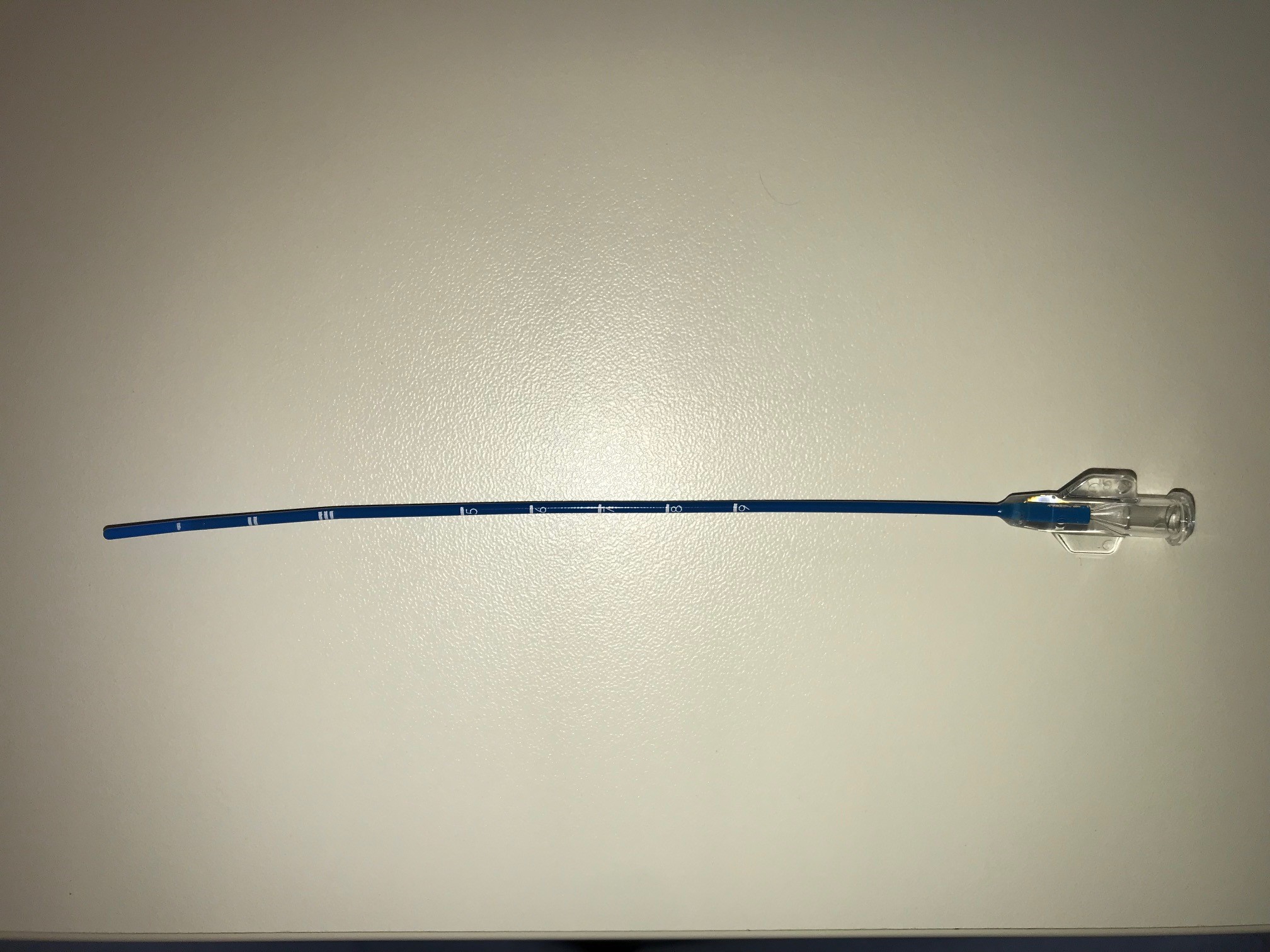


Figure 2. LISAcath® *(1,7x130mm REF 145872-01 Chiesi Farmaceutici S.p.a. Italy*) (Photo taken by Lorenz Auer-Hackenberg)

The LISAcath® is made from more rigid plastic. It is completely straight but can be slightly bent to be shaped into a curve and facilitate intubation which can be performed without the use of additional forceps. Centimeter markers from the tip of the tube serve as index to estimate intubation depth. The device has a valid CE grading and clearance for the clinical use in neonates. This technique is currently already used by the study site’s staff members during a test phase in 2018.

All physicians at the study site are well trained in both techniques. At June/July 2018 the LISAcath® will be made available commercially and due to very positive experiences during the initial testing the Division of Neonatology will change the current SOP and the use of LISAcath® for surfactant administration in LISA setting will become standard of care.

#### Primary endpoint:

Successful intratracheal tube placement.

#### Null hypothesis

Time of laryngoscopy during LISA protocol with both catheters (nasogastric tube and LISAcath®) is equal.

#### Alternative hypothesis:

Time of laryngoscopy during LISA protocol is shorter with LISAcath® application.

## Sample size

Sample size estimation will be done for primary outcome, namely the procedure duration.

In a pilot study with 40 students, procedure durations for feeding tube with Magill forceps (n1 = 21) and LISAcath® (n2 = 19) were recorded (see Table 1). As Rigo et al. (2017) showed, for neonatologists the procedure durations for feeding tube with Magill forceps and 13cm rigid angio catheter (similar to the LISAcath®) are below 90 seconds for both used simulators (Neonatal Intubation Trainer and ALS Baby Trainer)^8^. The results of our pilot study show, that some of the students needed much more than 90 seconds, which might be due to insufficient experience in applying the procedure. Therefore, students with procedure time >120 seconds were excluded from sample size estimation, since these would not be representative for the study sample. This decreases the sample size of the pilot study down to 35 (n1 = 17, n2 = 18) students. This cohort served as the basis for the sample size estimation that is reported in the sequel. All calculations were done for alpha = .05 and beta = .80. A time difference of 30 seconds is considered as a relevant effect (Δ), and 1:1 group allocation is assumed. We consider a laryngoscopy duration difference of 30 seconds as relevant effect because prolonged (i.e. more than 30 seconds) laryngoscopy is considered to be associated with significant discomfort for the baby during our SOP protocol. The underlying variance was assumed to be s² = 0.22 (i.e., the sample variance from the pilot study). Sample sizes are calculated for two different scenarios, depending on whether or not the assumption of a normal distribution is made. For normally distributed data, the sample size is estimated using the Guenther-Schouten-formula, that is, the classical t test formula with a correction for small samples (Schouten, 1999). This results in an estimated minimum sample size of 32 (n1 = 16, n2 = 16). In case of not non-normal data, the Wilcoxon-Mann-Whitney test is used instead of the t-test, which yields a total sample size of at least 14 (n1 = 7, n2 = 7). The calculations were done by “WMWssp”-package in R (Version 3.4.3), which is an implementation of the procedure that was proposed in Happ et al. (2019) ^11,12^

### Data acquisition

After inclusion in the study, a member of the study team will attend the LISAprocedure.

### Time of laryngoscopy

During the procedure, members of the study team will take the time from the beginning of laryngoscopy (as soon as the laryngoscope gets in contact with the baby) until its removal. If more than one laryngoscopy attempts are needed before the physician in charge of the procedure is satisfied, the time of each attempt will be added up. Data will be written on a predefined case-report-form by the study personal (see Appendix 3) If no written signed informed consent is available or none can be obtained within 24h, the collected data will be deleted.

#### Hypothesis:

Laryngoscopy and intratracheal catheter placement is significantly faster with LISAcath® device.

### Vital signs

During the procedure, transcutaneous oxygen saturation (SpO2; %) and heart rate (HR, beats per minute) will be continuously recorded via Phillips IntelliVue X2 monitor. Vital signs are continuously recorded by members of the study team and entered into a predefined case report form (see Appendix 3). After informed consent is obtained (see above) the child’s vital parameters can be extracted from the software.

#### Hypothesis:

Laryngoscopy and intratracheal catheter placement with LISAcath® device causes less depression of heart rate and transcutaneous oxygen saturation.

#### Study design:

The study will be conducted as a post marketing study between November 1^st^ 2019 until April 30^th^ 2020.

## Data management

Data from the case-report-form will be entered in digital spread sheets at the study site. Upon enrolment, every patient will be assigned a consecutively generated two-digit identification number that serves as a label for a patient's dataset record as well as his or her case report form. The patient’s full name, contact details or any other identification code will not be written on the case-report-form or entered in the electronic dataset. If a parent or caregiver chooses to opt out of the study, the case-report-form will be destroyed and no electronic record will be entered.

## Analysis/Statistics

Data will be analyzed using R (Version 3.4.3). Descriptive statistics will be done for epidemiological data.

Primary outcome: For normally distributed data, the two-sample t-test will be applied for examining differences regarding the duration of the intervention (primary outcome). In case of variance inequality, the Welch-Satterthwaite t-test will be used. If the data is not normally distributed, corresponding non-parametric tests (e.g. Wilcoxon-test) will be applied.

Secondary outcomes: Will be analysed by appropriate methods for repeated measurements (heart rate and oxygen saturation). Further exploratory analysis of the secondary outcomes will be conducted using regression models.

All statistical tests will be carried out at the five percent level. No adjustment for multiple testing will be done because there is only one primary outcome. As well no correction for secondary outcomes will be applied due to the exploratory style of the Analysis. For all outcomes 95%-Confidence Intervals will be calculated as well.

## Ethical considerations

All physicians participating in the study adhere to the declaration of Helsinki and have valid good clinical practice (GCP) certification. Both studied techniques (nasogastric tube and LISAcath®) are well established methods for surfactant application at our site. All doctors are well trained in both techniques. Randomly assigning participants to a certain technique, for the purpose of this study, does not pose additional risks or health hazards.

The protocol does not influence the physician’s choice of surfactant administration method. Every physician in charge of providing care to a newborn infant can chose to switch between methods or even disregard the institution’s SOP for LISA if she or he considers a different course of action or a different method to be more appropriate for the child. The patient will be enrolled in our database as long as either method is used according to LISA standards. The feeding tube inserted with Magill forceps remains the principle vehicle of surfactant delivery in international studies^5^. Currently the feeding tube has no CE certification for endotracheal drug application and we include a thorough risk assessment for this circumstance. In any case, the physician who supervises cardiocirculatory adaptation and performs the LISA-procedure can alter the course of treatment as needed, regardless of the study or its protocol.

## Eingehaltene Normen

Im Rahmen der Studie wurden folgende Normen EN ISO eingehalten:

- Prüfplan/Studiendesign etc: EN ISO 14155
- Eingehaltene Normen LISAcath(r):
  - wie beschrieben in der Technical File: ISO 9001:2008; EN ISO 11135-1, EN ISO 11737-1, EN ISO 11737-2) QMS Certificate ISO 13485
- Eingehaltene Normen NGTube:
  - Wie beschrieben in der Technical File: ISO13485

**Safety Reporting**

An Adverse Event (AE) is “any untoward medical occurrence in a patient or clinical trial neonate administered a medicinal product and which does not necessarily have a causal relationship with this treatment”.

Therefore, an AE can be any unfavourable and unintended sign (including abnormal heart rates or laboratory results), symptom, or disease temporally associated with the use of a medicinal product, whether or not considered related to products under investigation.

A Serious Adverse Event (SAE) is any untoward medical occurrence or effect that at any dose falls in one or more of the following categories:

a) led to a death, injury or permanent impairment to a body structure or a body function.

b) led to a serious deterioration in health of the subject, that either resulted in:

- a life-threatening illness or injury, or

- a permanent impairment of a body structure or a body function, or

- in-patient hospitalization or prolongation of existing hospitalization, or

- in medical or surgical intervention to prevent life threatening illness

c) led to foetal distress, foetal death or a congenital abnormality or birth defect or tumor development.

Examples:

In case a.) death is considered as an outcome. It is the cause of death that should be recorded as the adverse event with the exception of “sudden death” where no cause has been established; in this latter instance, “sudden death” should be regarded as the adverse event and “fatal” as its reason for being serious. Life-threatening refers to an event in which the subject was at risk of death at the time of the event (e.g., major bleeding). The term does not refer to an event which hypothetically might have caused death if it were more severe. Hospitalization refers to a situation whereby an AE is associated with unplanned overnight formal admission into hospital, usually for purpose of investigating and/or treating the AE. As these premature neonates will be hospitalized at inclusion and during the first few weeks of the study the sections of the above definition relating to hospital admissions will be of less relevance during that inpatient period. The term significant disability should be viewed as any situation whereby an AE has a clinically

important effect on the subject’s physical or psychological well-being to the extent that the subject is unable to function normally. Medical and scientific judgment should be exercised in deciding whether an event is serious because medically significant.

Any suspected transmission via a medicinal product of an infectious agent is also considered a serious adverse reaction.

Examples of SAE

- Pharyngeal/laryngeal and pulmonary hemorrhage
- Severe bradycardia requiring medical intervention
- Allergic/topical reactions to material
- Perforation (intraoral, laryngeal or pulmonary)
- Pneumothorax requiring medical intervention
- Dislocation of tube in to the esophagus after initial correct placement

Recording:

AEs occurring during the course of the study must be documented. Moreover, if the Adverse Event is serious, the Serious Adverse Event must be recorded and the sponsor has to be informed immediately.

It is responsibility of the Investigator to collect all adverse events (both serious and non-serious).

As a general rule, the recording period for Adverse Events is the period starting from the neonate’s birth (if the Informed consent is signed before birth) until

the subject’s study participation ends after 24h.

All SAEs have to be documented by the sponsor and immediately reported according to § 42 (8) and § 70 of the Austrian Medical Devices Act to the competent authority (AGES) and the competent authorities of other countries within the European Union where the study is conducted. Please note, there are no further specified reporting timelines in the Austrian Medical Devices Act, here you can follow the MEDDEV 2.7/3 guideline requirements.
Serious adverse events that result in immediate risk of death, serious injury or illness, need to be reported without delay, at the latest within 2 calendar days to the BASG, all other events within 7 calendar days. All SAEs must be reported using the templates provided by AGES. The Investigator must notify the affected ethics committee without delay of any serious side effects and any serious adverse events during the clinical trial (MPG § 61). The clinical investigator must inform the sponsor of any medical device effects and any serious adverse events during the clinical trial (MPG § 64, (5)). Furthermore a continuous reporting form (tabular listing = line listing) F_I287 (SAE report table) must be maintained for all SAEs (occurring abroad or in Austria), and the BASG must immediately be notified if new SAEs or changes to or additions to previously reported SAEs occurred.

**Declaration**:

*Es wird bestätigt dass bei der Anwendung des Prüfproduktes/Komparators (inkl. Zubehör) im Rahmen der Studie alle in den vorgelegten Gebrauchsanweisungen festgelegten Kriterien (wie Verwendungszweck, Kontraindikationen, Risiken, Warnhinweis und Vorsichtsmaßnahmen) berücksichtigt werden*

*Der Komparator wird mit Ausanhme der Punkte, die Gegenstand der klinischen Prüfung sind, den grundlegenden Anforderungen der Richtlinige entsprechen.*

# References

1. Fujiwara, T. *et al.* Surfactant replacement therapy with a single postventilatory dose of a reconstituted bovine surfactant in preterm neonates with respiratory distress syndrome: final analysis of a multicenter, double-blind, randomized trial and comparison with similar trials. The Surfactant-TA Study Group. *Pediatrics* **86,** 753–64 (1990).

2. Hobar, J. D. *et al.* A Multicenter Randomized, Placebo-Controlled Trial of Surfactant Therapy for Respiratory Distress Syndrome. *New Engl J Medicine* **320,** 959–965 (1989).

3. Suresh, G. K. & Soll, R. F. Overview of Surfactant Replacement Trials. *J Perinatol* **25,** 7211320 (2005).

4. Ammari, A. *et al.* Variables Associated with the Early Failure of Nasal CPAP in Very Low Birth Weight Infants. *J Pediatrics* **147,** 341–347 (2005).

5. Kribs, A., Pillekamp, F., Hünseler, C., Vierzig, A. & Roth, B. Early administration of surfactant in spontaneous breathing with nCPAP: feasibility and outcome in extremely premature infants (postmenstrual age ≤27 weeks). *Pediatr Anesth* **17,** 364–369 (2007).

6. Härtel, C. *et al.* Less invasive surfactant administration and complications of preterm birth. *Sci Rep-uk* **8,** 8333 (2018).

7. Fabbri, L. *et al.* Five‐country manikin study found that neonatologists preferred using the LISAcath rather than the Angiocath for less invasive surfactant administration. *Acta Paediatr* **107,** 780–783 (2018).

8. Rigo, V., Debauche, C., Maton, P., Broux, I. & Laere, V. D. Rigid catheters reduced duration of less invasive surfactant therapy procedures in manikins. *Acta Paediatr* **106,** 1091–1096 (2017).

9. Dargaville, P. A. *et al.* The OPTIMIST-A trial: evaluation of minimally-invasive surfactant therapy in preterm infants 25–28 weeks gestation. *Bmc Pediatr* **14,** 213 (2014).

10. Fenton, T. R. & Kim, J. H. A systematic review and meta-analysis to revise the Fenton growth chart for preterm infants. *Bmc Pediatr* **13,** 59 (2013).

11. Happ, M., Bathke, A. C. & Brunner, E. Optimal sample size planning for the Wilcoxon‐Mann‐Whitney test. *Stat Med* **38,** 363–375 (2019).

12. Schouten, H. J. Sample size formula with a continuous outcome for unequal group sizes and unequal variances. *Stat Med* **18,** 87–91 (1999).

13. Roberts Ch. *et al.* Randomisation methods in controlled trials Chris Roberts, David Torgerson. BMJ **317**, 1301-1310 (1998)

# Appendices

### Student questionnaire

1. Gender: male/female
2. Age: 18-20 years; 20-22 years; 22-25 years; 25 years or older
3. Study year: first, second, third, fourth
4. Previous intubation experience: none; yes (emergency medicine class, paramedic training, others)
5. Previous successful intubation attempts: none; yes (on a dummy; during real-life resuscitation, other)
6. Handedness: right; left
7. Preferred method: LISAcath®; nasogastric tube
8. Assigned method: LISAcath®; nasogastric tube (filled out by investigators)
9. Time needed (filled out by investigators)

### LISA SOP (german)

LISA Erstversorgung

FG <SSW 28+0

Personalbedarf:

Einling: 2 Ärzte, 2 Pflegekräfte

Zwillinge: 3 Ärzte, 3 Pflegekräfte

Drillinge: 4 Ärzte, 4 Pflegekräfte

…

Mit Mundschutz/ Haube und sterilen Handschuhen

Vorbereitungen:

Erstversorgungsplatz (pro Kind):

- Inkubator: maximal gewärmt, mit 2-3 Windeln ausgelegt plus für Kind passende Windel bestückt.
- Neo-Wrap ausgelegt
- Augenschutz für Umgebungslicht
- Magensonde (5F) oder Schlürfsonde (6F)
  - mit Spritzen zum Aspirieren (klein und groß) und Fixierung
- Hydrosun oder Heizstrahler vom Inkubator eingeschalten
- Erstversorgungswagen:
  - Monitor mit Sättigung und EKG bestückt.
  - T-Stück Resuscitator mit beheizbaren Schläuchen und Heizung:
    - 15 l/min, 40% O2 und PEEP 20-23 mbar
    - kleineste Maske
    - Auqa für Heiztopf (feuchte warme Schläuche können 24h ungenützt hängen)
    - Druckluft+O2-Schläuche sind an der Wand angesteckt, sollen sich aber für den Transport an die Station an die transportablen Flaschen anschließen lassen
    - Absaugung auf -140 kPas
  - transportable O2- und Druckluftflaschen
  - Beatmungsbeutel (in Reserve für Notfall)
  - 4 Spritzenpumpen/Perfusoren
    - Infusion/Medikamente (Dosierung laut Gewichtstabelle)
      - Hauptinfusion (Elomel –Semiton)
      - Standacillin (50 mg/kg)
      - Coffeinzitrat (20 mg/kg)
      - Refobacin (5 mg/kg)
      - Diflucan (3 mg/kg)
    - Alle mit Leitungen und Verteiler verbunden, Klemmen geöffnet

Erstversorgungstisch:

- Tubus für Rachen-CPAP (Laut Gewichtstabelle)
  - Fixierung
  - Tupfer zum Entfetten+Pflesterlöser
- Venenzugang:
  - 2x Venflon (1x gelb, 1x violett)
  - Spülung
  - Fixierung
  - Tupfer zum desinfizieren + Octenisept
  - Astrupspritze, Spritze für Blutkultur, 2 Nadeln, Blutkultur
- Surfactant (200 mg/kg) in Spritze aufgezogen
- LISAcath oder alternativ Magensonde für Surfactant
  - Markierung der Magensonde mit Edding 3000 (wasserfester dicker Stift) mittig zwischen proximalem Loch und 1. aufgedruckter Markierung unter sterilen Bedingungen (Haube, Maske und sterile Handschuhe).
- Laryngoskop (schmaler 0‘er Spatel)
- kleinste Magill-Zange (nur bei Magensonde notwendig)

Für die Position:

- binasaler CPAP (PEEP 10 mbar)
- Arterien Flush (30 IE Heparin auf 30 ml NaCl 0,9%; Laufrate 0,2 ml/h)
- Metalline als Unterlage und Lagerungsbehelf
- Lagerungsbehelfe
- 2. Neo-Folie zum Abdecken des Kindes

Überprüfung der Vorbereitungen mittels Abarbeiten der Checkliste

Erstversorgung:

- Deckel des Inkubators unmittelbar vor Eintreffen des Kindes anheben oder abnehmen und alle Seitenwände nach unten klappen.
- Hydrosun beleuchtet die Mitte des Inkus.
- Kind wird vom Tuch der Hebamme genommen und in die Neo-Folie geschlagen.
- Lagerung in Re-Seitenlage, quer im Inkubator im Lichtstrahl der Hydrosun oder unter Heizstrahler, Arzt am Kopf, Pflege bei den Füßen des Kindes.
- Arzt setzt Perivent-Maske auf und Appliziert den PEEP von 20-23mbar.
  - Fehlende Eigenatmung kann zumindest für 2 Minuten akzeptiert werden.
- Pflege befestigt Sättigungssensor am rechten Arm, legt Temperaturfühler unter das Kind, deckt die Augen des Kindes gegen Umgebungslicht und den Sättigungssensor gegen das Infrarotlicht der Hydrosun ab und lagert das Kind mit angezogenen Beinen und Händen im Bereich des Mundes.
- Zieltemeratur 37,0°C - 37,5°C
- Abwarten bis Eigenatmung etabliert und stabile Sättigung vorliegt.
  - Ziel:
    - 1 Min: Hf >100/min; SaO2 um 50%
    - 5 Min: Hf >130/min; SaO2 >60%
    - 10 Min: Hf >130/min; SaO2 >80%
    - 20 Min: Hf >130/min; SaO2 >90%
- Maskenbeatmung NUR bei Bradycardie UND ungenügender Sättigung. Bei Vollnarkose der Mutter großzügiger!
- FiO2 wird nur bei nicht erreichen dieser Ziele, dann aber großzügig erhöht und genauso zügig wieder reduziert.
- Nach Stabilisierung oder wenn trotz steigender O2 Zufuhr keine Stabilisierung erzielt werden kann Einführen des Rachen-CPAP’s und umstecken des T-Stücks.
  - Am Rachen-CPAP anfänglich noch 2. Nasenloch und Mund vorsichtig dicht halten, nach Stabilisierung muss versucht werden, ob das Kind den CPAP auch mit offenem Mund und Nasenloch toleriert.
- Magensonde durch den Mund verlegen und Absagen der Luft aus dem Magen (Schlürfsonde kontinuierlich).
- Zeitpunkt des Abnabelns kann individuell gestaltet werden, hat aber ganz sicher keine Priorität, bei der Gelegenheit kann dem Kind auch die Windel angelegt werden.
- Nach neuerlicher Stabilisierungsphase Platzwechsle von Arzt und Pflege.
- Verlegen eines venösen Zuganges möglichst am Bein des Kindes (V. saphena magna am rechten Innenknöchel!).
  - Blutabnahmen:
    - Blutkultur
    - Astrup
- Start mit Hauptinfusion (Elomel-Semiton) laut Gewichtstabelle.
- Start mit den Medikamenten:
  1. Standacillin als Bolus i.v..
  2. Coffeinzitrat innerhalb von 5 Minuten als KI.
  3. Refobacin innerhalb von 20 Minuten als KI.
  4. Diflucan innerhalb von 20 Minuten als KI.
- Neuerlicher Platzwechsle von Arzt und Pflege.
- Nach Beendigung der Coffeininfusion umlagern des Kindes auf Rückenlage, rechts und links durch „Windelrollen“ stabilisiert.
- Spitze des LISAcath leicht anwinkeln oder Magensonde in Magill-Zange einklemmen (in einem Winkel von ca. 120° 1cm vor der Spitze entfernt) und so fixieren/halten oder hinlegen, dass der Arzt sie ohne Blickwendung greifen kann.
- Einstellen des Larynxeinganges/Epiglottis und Vorschieben von LISAcath bis Markierung II oder Magensonde bis zur Markierung.
  - Nach erfolgreicher Verlegung der Sonde ZUERST Magill-Zange unter Sicht und dann erst Laryngoskop aus dem Kind entfernen. Dabei Sonde immer gut fixieren!
- LISAcath oder Magensonde gemeinsam mit CPAP Tubus und Magensonde mit linker Hand fassen
- Applikation des Surfactant über 2-3 Minuten.
  - Parallel dazu Absaugen der Luft aus dem Magen um eventuelle Fehlapplikation von Surfactant in den Magen rasch zu erkennen.
  - Wenn Magensonde disloziert, diese zwischenzeitlich neu plazieren!
- Bei geringem Sättigungsabfall Maskenbeatmung über Perivent.
- Bei starkem Sättigungsabfall und Bradycardie Entfernen der Magensonde aus der Trachea und Stabilisierung des Kindes mittels Maskenbeatmung und/oder PEEP Applikation. Danach neuerliches Verlegen der Magensonde und restlichen Surfactnat verabreichen.
- Nach Surfactantapplikation überschüssigen Surfactant aus dem Mund/Nasen/Rachenraum absaugen.
- Stabilisierung der Eigenatmung nach der Surfactantgabe (Immer etwas höherer O2 Bedarf als davor), dafür eventuell in Li-Seitenlage bringen.
- Bonden Haut auf Haut mit der Mutter noch im Sectio-OP oder Kreißsaal für möglichst 30 Minuten
  - Bei stabilem Kind ev. auch im Aufwachraum der Gyn.
- Initiieren der Kollostrummassage
- Transport des Kindes an die Station am Rachen-CPAP.
  - Dafür Schließen des Inkubators und Umstecken der Wandversorgung auf die transportable Flaschenversorgung.

Nach Erstversorgung an der Position:

- Messen von Kopfumfang, Länge und Gewicht des Kindes.
- Durchführung Schädel-US
- Aufsetzen der CPAP Haube.
- Entfernen vom Rachen-CPAP und Anbringen des binasalen CPAPs.
  - Bei dieser Gelegenheit Fotos vom Kind ohne Atemunterstützung anfertigen (Infant Flow kann bevor er endgültig fixiert ist kurz zum „Abdrücken“ des Fotos weggehalten werden!)
- Umlagern des Kindes aus der Folie auf die Metalline (Nest) und mit frischer Folie abdecken.
  - Wechsel der Metalline wie „normale“ Bettwäsche im Inkubator, Folie nur bei Verschutzung.
  - Folie und Metalline zumindest 7 Tage oder so lange bis Verhornung der Haut abgeschlossen ist belassen.
- Verlegen eines (peripheren) arteriellen Zuganges.
- Verlegen eines zentralen Zuganges nur bei Katecholaminbedarf sofort, sonst frühestens am 2. Lebenstag oder wenn der 1. Venflon kaputt wird.
- Weitere Pflege laut Standart!
  - Minimal Händling!
  - Minimal Ärztling!
  - Waschen des Kindes in der Regel erst nach 6-7 Tagen notwendig.
  - …

## 3. Case-Report-Form

| Study ID | ______ | (chronologisch) |
| --- | --- | --- |
| Randomisierung zu welchem Kathetersystem?  1 = Magensonde  2 = LISAcath® |  |  |
|  |  |  |
| Informed Consent unterschrieben? | Vorhanden O | Nicht vorhanden O |
| Gestationsalter | _____ SSW | (z.B. 25+3/7) |
| Geburtsgewicht | _________ g | (z.B. 1020g) |
| Wurde LISA SOP eingehalten? | Ja O | Nein O |
| Welche Methode wurde verwendet?  1 = Magensonde  2 = LISAcath® |  |  |
| Wurde die zugewiesene Methode gewechselt? | Ja O | Nein O |
| Wenn Ja: Warum (Kommentar) | ____________________________________ | |
| Datum d. Erstversorgung | ___. ___. ___ | (z.B. 13.08.2019) |
| Zeitpunkt d. Erstversorgung | ____ : ____ | (z.B. 14:30) |
|  |  |  |
| Erstversorgung durchführender Arzt | _________________ |  |
|  |  |  |
| Benutzte Methode | Magensonde O | LISAcath® O |
|  |  |  |
| Vitalparameter VOR Laryngoscopie (L.) |  |  |
| Herzfrequenz | _____ /min | (z.B. 150/min) |
| Sauerstoff Sättigung | _____ % | (z.B. 91%) |
|  |  |  |
| Vitalparameter BEGINN L. |  |  |
| Herzfrequenz | _____ /min | (z.B. 150/min) |
| Sauerstoff Sättigung | _____ % | (z.B. 91%) |
|  |  |  |
| Vitalparameter 10s nach Beginn L. |  |  |
| Herzfrequenz | _____ /min | (z.B. 150/min) |
| Sauerstoff Sättigung | _____ % | (z.B. 91%) |
|  |  |  |
| Vitalparameter 20s nach Beginn L. |  |  |
| Herzfrequenz | _____ /min | (z.B. 150/min) |
| Sauerstoff Sättigung | _____ % | (z.B. 91%) |
|  |  |  |
| ZEITDAUER L. | ______ sek. | (z.B. 35 Sekunden) |
|  |  |  |
| Vitalparameter nach Beendigung L. |  |  |
| Herzfrequenz | _____ /min | (z.B. 150/min) |
| Sauerstoff Sättigung | _____ % | (z.B. 91%) |
|  |  |  |
| Vitalparameter 5s nach Beendgung L. |  |  |
| Herzfrequenz | _____ /min | (z.B. 150/min) |
| Sauerstoff Sättigung | _____ % | (z.B. 91%) |
|  |  |  |
| Vitalparameter 10s nach Beendigung L. |  |  |
| Herzfrequenz | _____ /min | (z.B. 150/min) |
| Sauerstoff Sättigung | _____ % | (z.B. 91%) |
|  |  |  |
|  |  |  |

Kommentar: (Verletzung während Laryngoskopie)?
